# Supplementary material for: Contraception and Hormone Replacement Therapy in Healthy Carriers of Germline BRCA1/2 Genes Pathogenic Variants: Results from an Italian Survey
Source: Cancers (Basel). 2022 Jul 15;14(14):3457. doi: 10.3390/cancers14143457 (PMC9315728; doi:10.3390/cancers14143457)
Supplement: Supplementary file 1 [file cancers-14-03457-s001.zip › cancers-1766013-supplementary.pdf]

Survey on contraception and hormone replacement therapy in healthy carriers of germline BRCA1/2 genes pathogenic variants (English translation of the questionnaire used)

The purpose of this research project is to investigate contraception and hormonal replacement therapy prescription and uptake in healthy carriers of BRCA 1-2 pathogenetic variants.

Your participation in this research study is voluntary. You may choose not to participate. If you decide to participate in this research survey, you may withdraw at any time. If you decide not to participate in this study or if you withdraw from participating at any time, you will not be penalized.

The procedure involves filling out an online survey that will take approximately 15 minutes. Your responses will be confidential, and we do not collect identifying information such as your name, email address, or IP address. The results of this study will be used for scholarly purposes and shared, in aggregate form only and without any identifying information, to educate the population on the issue.

If you have any questions about the research study, please contact segreteria@abrcacadabra.it or actocampania@gmail.com

1. By agreeing, you certify that you are at least 18 years old and you consent to the participation.

- Agree
- Do not agree

2. How old are you?

3. What is your education level?

- Elementary or middle school
- High school
- College or higher

4. Do you have any children?

- Yes
- No

5. How old you were when you received the diagnosis of *BRCA* pathogenic variant carrier?

6. Are you a carrier of a pathogenetic variant of:

- *BRCA1*
- *BRCA2*

7. Where is the hospital/clinic located where you are being treated:

- North Italy
- Central Italy
- South Italy
- Islands

8. Is a fertility service available in the hospital/clinic where you are being followed:

- Yes
- No

9. Have you undergone risk-reducing surgery?

- Mastectomy only
- Adnexectomy only
- Both
- None
- Only bilateral salpingectomy

10. If you underwent bilateral mastectomy, at what age?

11. If you underwent bilateral adnexectomy, at what age?

12. If you could go back in time:

- I would make the same choices regarding risk-reducing surgery
- I would undergo risk-reducing surgery earlier
- I would not undergo risk-reducing surgery
- Other (explain)

13. How relevant is sexual quality of life after *BRCA* carrier status detection? 0= not important at all, 10= extremely important

14. Do you feel like there was enough attention on sexual quality of life during your visits/counseling as a *BRCA1/2* pathogenic variant carrier? 0= not at all, 10= yes, a lot of attention

PART A - contraception

Do you feel like you have received enough information about contraception during your visits (with any healthcare provider)?

- Yes
- No

Have you ever discussed hormonal contraception specifically with your gynecologist?

- Yes, because I asked
- Yes, prompted by the physician
- No, because I have never needed it
- No, because I thought I could not use it for my increased cancer risk

What type of contraception have you used after *BRCA* carrier diagnosis?

- Barrier method only (condom)
- Combined hormonal contraception (pill, patch, vaginal ring)
- Progestin pill
- Copper IUD
- LNG-IUS

- None
- Other (explain)

The use of hormonal contraception in *BRCA* pathogenic variant carriers who have never had cancer:

- Increases ovarian cancer risk
- Decreases ovarian cancer risk
- Does not alter ovarian cancer risk
- Increases breast cancer risk
- Decreases breast cancer risk
- Does not alter breast cancer risk

After discussing hormonal contraception with the physician:

- I felt reassured
- My doubts about hormonal contraception safety increased
- Nothing changed

PART B - menopause

How old were you at menopause?

Your menopause was:

- Spontaneous
- Consequent to risk-reducing surgery

From 0 (the worst possible) to 10 (the best possible), which vote would you give to your sexual quality of life after menopause?

Do you feel like you have received enough information about hormone replacement therapy (HRT) during your visits (with any healthcare provider)?

- Yes
- No

Have you ever discussed HRT specifically with your gynecologist?

- Yes, because I asked
- Yes, prompted by the physician
- No, because I have never needed it
- No, because I thought I could not use it for my increased cancer risk

What were the menopause symptoms for which you felt the need of specific treatment?

- Pain during intercourse
- Hot flashes
- Mood swings
- Prevention of long-term effects (bone, CVD, cognitive function)
- All
- None
- Other (explain)

What type of menopause therapies have you used after BRCA carrier diagnosis?

- o Combined hormonal therapy (pill, transdermal gel or patch, etc.)
- o Tibolone
- o Estrogens/Bazedoxifene
- o Vaginal estrogens
- o Only vaginal laser (CO2 or erbium)
- o None
- o Other (explain)

If you never used HRT, what was the reason:

- o I never needed it
- o The physician told me it was contraindicated in BRCA mutation carriers
- o I feared using hormones because of the cancer risk
- o I was scared of other possible adverse effects
- o I used it

Have you used non-hormonal remedies for menopause symptoms?

- o Diet, exercise
- o Phytoestrogens (ex. soy)
- o Vitamins/minerals
- o Homeopathy
- o Acupuncture
- o Yoga/relaxation techniques
- o Vaginal moisturizers—lubricants
- o Vaginal laser
- o None
- o Other (explain)

The use of HRT in BRCA pathogenic variant carriers who have never had cancer:

- o Increases ovarian cancer risk
- o Decreases ovarian cancer risk
- o Does not alter ovarian cancer risk

- o Increases breast cancer risk
- o Decreases breast cancer risk
- o Does not alter breast cancer risk

After discussing hormonal contraception with the physician:

- o I felt reassured
- o My doubts about hormonal contraception safety increased
- o Nothing changed

Do you have other comments/observations?

Thank you for your participation.
